# Efficacy and Safety of CSF Shunting for Idiopathic Normal Pressure Hydrocephalus: A Systematic Review and Meta-Analysis

**DOI:** 10.7759/cureus.107776

**Published:** 2026-04-27

**Authors:** Mohammad Haroon Hafeez, Shahzad Muzaffar, Muhammad Waqas Umer, Zunaira T Ramzi, Sanya Raza, Syed Jawad Haider, Samar Hameed, Mufaya Nganzi, Zara Chaudhry, Shakil Ahmad, Zoraiz Ahmed

**Affiliations:** 1 Neurosurgery, Punjab Institute of Neurosciences, Lahore General Hospital, Lahore, PAK; 2 Neurological Surgery, Chaudhry Muhammad Akram Teaching and Research Hospital, Lahore, PAK; 3 Neurological Surgery, Nishtar Medical University, Multan, PAK; 4 Neurology, Akhtar Saeed Medical and Dental College, Lahore, PAK; 5 Neurology, Liaquat University of Medical and Health Sciences, Jamshoro, PAK; 6 Public Health and Epidemiology, Walden University, Minneapolis, USA; 7 Medicine and Surgery, Sialkot Medical College, Sialkot, PAK; 8 Surgery, Authentic Hospital Ltd., Dhaka, BGD; 9 Ophthalmology, Ispahani Islamia Eye Institute and Hospital, Dhaka, BGD; 10 Medicine, Shifa International Hospitals Limited, Islamabad, PAK

**Keywords:** cerebrospinal fluid shunting, gait velocity, idiopathic normal pressure hydrocephalus, lumboperitoneal shunt, ventriculoperitoneal shunt

## Abstract

Idiopathic normal pressure hydrocephalus (iNPH) is a potentially reversible neurological condition characterized by gait disturbance, cognitive impairment, and urinary dysfunction. CSF shunting remains the standard treatment; however, reported efficacy and complication rates vary across studies. This systematic review and meta-analysis aimed to evaluate the effectiveness and safety of CSF shunting in patients with iNPH based exclusively on evidence from randomized controlled trials (RCTs). The review was conducted in accordance with Preferred Reporting Items for Systematic Reviews and Meta-Analyses (PRISMA) guidelines. Literature searches were performed in PubMed, ScienceDirect, and the Cochrane Library for English-language RCTs published since 2010. Eligible studies included patients aged ≥60 years with radiologically confirmed iNPH who underwent ventriculoperitoneal or lumboperitoneal shunting. Primary outcomes included changes in modified Rankin Scale (mRS) scores and gait velocity. Secondary outcomes included cognitive and urinary measures, as well as adverse events. Random-effects models were applied. Three RCTs comprising 210 participants were included. CSF shunting was associated with significant improvement in functional outcomes (MD, -0.75; 95% CI, -1.01 to -0.49) and gait velocity (MD, 0.21 m/s; 95% CI, 0.12 to 0.29), with low heterogeneity. No significant improvement was observed in cognitive or urinary outcomes. However, shunting was associated with an increased risk of subdural hematoma. CSF shunting provides meaningful functional and gait benefits in carefully selected patients with iNPH, although it is associated with an elevated risk of subdural hematoma. Careful patient selection and risk-benefit evaluation remain essential.

## Introduction and background

Idiopathic normal pressure hydrocephalus (iNPH) is a progressive neurological disorder characterized by the classic clinical triad of gait disturbance, cognitive impairment, and urinary incontinence, along with ventriculomegaly, or enlargement of the brain’s ventricular system, and normal CSF pressure on lumbar puncture [1,2]. Although iNPH is more common among older adults, its prevalence is likely underestimated due to overlap with other neurodegenerative conditions, such as Alzheimer’s disease and Parkinsonism. However, the available evidence remains limited and is partly based on case-level observations [3,4].

The pathophysiology of iNPH remains unclear, and some factors, such as impaired CSF absorption and altered intracranial compliance, are believed to contribute to ventricular enlargement and consequent neurological impairment [5]. The most common treatments for iNPH include CSF shunting, a surgical procedure that diverts excess CSF to another part of the body, often via ventriculoperitoneal or lumboperitoneal shunts, to re-route the fluid and alleviate symptoms [6]. Although shunting has been shown to improve gait, cognition, and urinary control, findings are mixed, and complications such as infection, overdrainage, and shunt malfunction remain critical concerns [6,7]. Moreover, issues related to patient selection and predictors of positive outcomes remain controversial, making clinical decision-making more difficult [8].

Given the heterogeneity of the existing evidence, a systematic synthesis is necessary to more accurately quantify the efficacy and safety of CSF shunting in iNPH. This systematic review and meta-analysis critically evaluates the available evidence from randomized controlled trials (RCTs), providing a more robust and clinically reliable estimate of treatment effects. By focusing on high-quality trial data, this study seeks to clarify functional outcomes, gait improvement, associated complications, and potential factors associated with treatment response, thereby supporting more informed clinical decision-making.

Although numerous studies have investigated CSF shunting in iNPH, much of the existing literature is based on observational or single-center designs, which may introduce bias and limit the strength of conclusions. In contrast, this review focuses exclusively on RCTs, representing the highest level of evidence, to provide a more rigorous and unbiased assessment of the effectiveness and safety of CSF shunting.

Objectives

The objective of this study is to determine whether CSF shunting can improve the clinical symptoms of iNPH, such as gait disturbance, cognitive impairment, and urinary incontinence. It also assesses the incidence and characteristics of complications associated with CSF shunting in patients with iNPH. The research also aims to explore potential factors associated with shunt surgery, including patient characteristics, shunt characteristics, and perioperative conditions. Finally, the results may provide evidence-based recommendations to guide clinicians in selecting and managing patients with iNPH.

## Review

This systematic review was performed according to Preferred Reporting Items for Systematic Reviews and Meta-Analyses (PRISMA) guidelines, and the review was registered on PROSPERO (CRD420261320650) [9].

Search strategy

We mainly used three databases: PubMed, ScienceDirect, and the Cochrane Library, for our literature search. A detailed search strategy was developed for each database using “AND,” “OR,” and other Boolean operators. The search strategy used on PubMed was: ‘((((((((Hydrocephalus) OR (Normal Pressure Hydrocephalus)) OR (idiopathic normal pressure hydrocephalus)) OR (iNPH)) OR (NPH)) AND (CSF Shunt)) OR (VP shunt)) OR (Ventriculoperitoneal Shunt)) OR (Lumboperitoneal shunt)’. We used ‘(((Normal Pressure Hydrocephalus) OR (idiopathic normal pressure hydrocephalus) OR (iNPH) OR (NPH)) AND ((CSF Shunt) OR (VP shunt) OR (Ventriculoperitoneal Shunt) OR (Lumboperitoneal shunt)))’ for ScienceDirect. For the Cochrane Library, the search strategy was ‘("normal pressure hydrocephalus"):ti,ab,kw OR ("idiopathic normal-pressure hydrocephalus"):ti,ab,kw AND (Ventriculoperitoneal Shunt):ti,ab,kw OR ("VP shunt"):ti,ab,kw OR ("lumboperitoneal shunt"):ti,ab,kw’. The search across all databases was limited to trials published after 2010 through February 2026 to ensure relevance to updated clinical practice because of advancements in surgical techniques, imaging modalities, and perioperative care for CSF shunting. Moreover, the reference lists of each included study were reviewed to identify any additional relevant articles.

Eligibility criteria

Studies were selected if the participants were aged >60 years, had NPH, had normal opening CSF pressure, and had an Evans index >0.3. Studies in which CSF shunting was the primary intervention were included. This could include either VP or LP shunting. To be included, studies also had to report on one of the primary outcomes, i.e., modified Rankin Scale (mRS) scores or gait velocity. We only included RCTs published in English after 2010.

We excluded articles involving patients below 60 years of age, patients with NPH due to secondary causes, and patients who did not undergo either of the above-described CSF shunts. Additionally, patients with an Evans index <0.3 were excluded. Studies that did not report any of the primary outcomes were excluded. Similarly, studies that were not RCTs or were not in English were excluded.

Screening, selection, and data extraction

We screened all retrieved articles for duplicates by importing them into EndNote. After removing duplicates, we excluded all articles deemed irrelevant based on their titles and abstracts. The remaining articles underwent a full-text review. The articles with available full texts were then screened against our predesigned selection criteria. Two reviewers carried out this whole process, and any issues regarding the inclusion or exclusion of an article were resolved with the help of a third reviewer. Additionally, external assistance was used for the methodology and results.

Two reviewers read the articles thoroughly and extracted the following data into an Excel sheet: author ID, age, male-to-female ratio, comorbidities, shunt types, outcomes, and details of interventions.

Quality assessment 

The risk of bias in the RCTs included in this review was assessed using the Cochrane ROB2 tool [10], which includes five domains. This step was conducted by one reviewer and then reviewed by another reviewer. Studies were classified as having a high risk of bias if some concerns were observed in two domains or a high risk of bias was observed in at least one domain. A study was classified as low risk if all domains had a low risk of bias. Similarly, a study with some concerns in one domain and low risk in all other domains was classified as having “some concerns.”

Data synthesis

RevMan 5.4 was used to analyze the collected outcome data. For continuous outcomes, mean differences with SDs were used under the random-effects inverse variance model. For dichotomous variables, risk ratios were calculated using a random-effects Mantel-Haenszel model, with 95% CIs. I² was used as a measure of statistical heterogeneity. Heterogeneity was considered high for values above 75%, moderate for 51-75%, mild for 26-50%, and low for I² below 25%. Forest plots were used to present the results. Subgroup analysis could not be conducted due to the limited data available. Similarly, we were unable to perform a sensitivity analysis due to the limited number of studies. A p-value below 0.05 was considered significant. Potential publication bias was not evaluated using funnel plots because of the small number of included trials (n < 10).

Results

**Figure 1:**
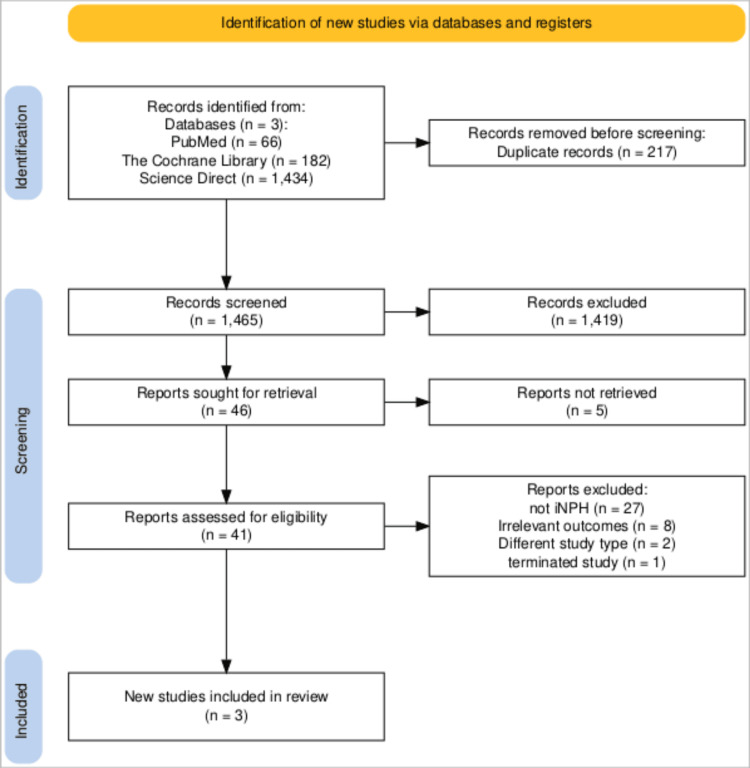
PRISMA flow diagram of the study selection process. PRISMA flow diagram illustrating the study selection process, including identification, screening, eligibility assessment, and final inclusion of randomized controlled trials. PRISMA: Preferred Reporting Items for Systematic Reviews and Meta-Analyses.

Our search across all databases yielded 1,682 articles, of which 217 were removed as duplicates. An additional 1,419 articles were removed as irrelevant during title and abstract screening. The full text of five articles was not available; therefore, these five articles were excluded at this stage. Our eligibility criteria were applied to 41 articles, and, in the end, we were left with three RCTs [7,11,12]. The entire screening and selection process is illustrated in Figure 1.

Characteristics of Studies

Table 1 shows the three RCTs, which included a total of 210 participants. The majority of the study populations were older patients, with a mean age of 73.1-76.1 years and consistent male predominance (51%-78%) across all studies. The Evans Index, used to standardize radiographic severity, showed remarkable consistency, with mean values ranging from 0.38 to 0.40, indicating considerable ventriculomegaly across all intervention groups. Although iNPH was the focus of all investigations, the surgical strategy differed between VP shunting in the Placebo-Controlled Efficacy in iNPH Shunting (PENS) trials and LP shunting in the SINPHONI-2 trial. In particular, SINPHONI-2 used an open-label, delayed-surgery control, whereas the PENS studies used an advanced double-masked, placebo-controlled design with “virtual-off” valve settings.

**Table 1:** Characteristics of included randomized controlled trials assessing cerebrospinal fluid shunting in idiopathic normal pressure hydrocephalus. Characteristics of the included studies, summarizing study design, sample size, patient demographics, intervention details, comparator groups, and reported outcomes. Source: References [7, 11, 12]. RCT: Randomized controlled trial; VP: Ventriculoperitoneal; LP: Lumboperitoneal; N: Number; MMSE: Mini-Mental State Examination; MoCA: Montreal Cognitive Assessment; H₂O: Water.

Characteristics	Luciano MG et al. (2025)	Kazui H et al. (2015)	Luciano M et al. (2023)
Study design	Double-blind, placebo-controlled RCT	Open-label RCT	Double-blind, placebo-controlled pilot study
Interventions	Open VP shunt: valve set to “open” setting, setting 4; 110 mm H₂O. Placebo: valve set to “virtual off,” setting 8; >400 mm H₂O, for 3 months.	Immediate LP shunt: surgery within 1 month. Postponed shunt: conservative therapy/exercises for 3 months, followed by LP shunt surgery.	Open VP shunt: valve set to setting 4, approximately 110 mm H₂O. Placebo: valve set to setting 8, “virtual off”; >400 mm H₂O, for 4 months.
Sample size, N	99; 49 open / 50 placebo	93; 49 immediate / 44 postponed	18; 9 open / 9 placebo
Age, mean ± SD	76.1 ± 6.3 open / 75.7 ± 6.6 placebo	75.3 ± 6.0 immediate / 74.9 ± 4.7 postponed	73.8 ± 5.5 open / 73.1 ± 6.9 placebo
Sex, male %	69% open / 62% placebo	51% immediate / 52% postponed	78% open / 67% placebo
Evans Index	0.36 ± 0.03	>0.3	0.37 ± 0.06
Cognition, MMSE/MoCA	MoCA: 20.7 ± 4.2 open / 21.4 ± 4.0 placebo	MMSE: 2.6 ± 5.8 immediate / 1.2 ± 2.8 postponed	MoCA: 1.63 ± 1.6 open / -0.13 ± 3.6 placebo
Shunt hardware	Codman Certas Plus with SiphonGuard	Codman-Hakim programmable valve with SiphonGuard	Codman Certas Plus with SiphonGuard

Risk of Bias

**Figure 2:**
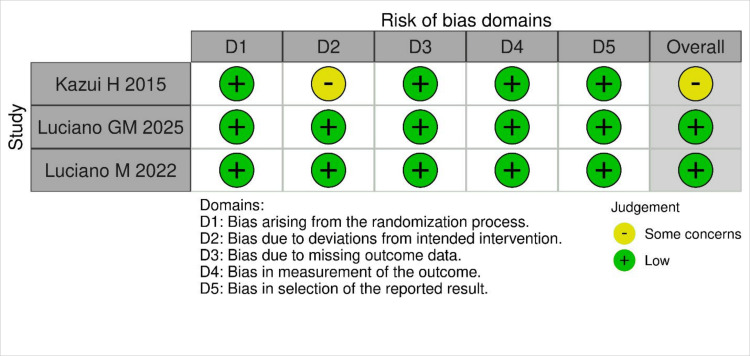
Risk of bias assessment of included studies. Risk of bias assessment of the included studies using the ROB 2 tool, showing overall bias judgments across different domains. Source: References [7, 11, 12].

The risk of bias assessed using ROB2 showed that one study had some concerns, while the other two studies had a low risk of bias. The study by Kazui H et al. raised concerns about deviations from the intended interventions, as participants were aware of the intervention. A summary of the risk of bias is provided in Figure 2 below.

Primary Outcomes

**Figure 3:**
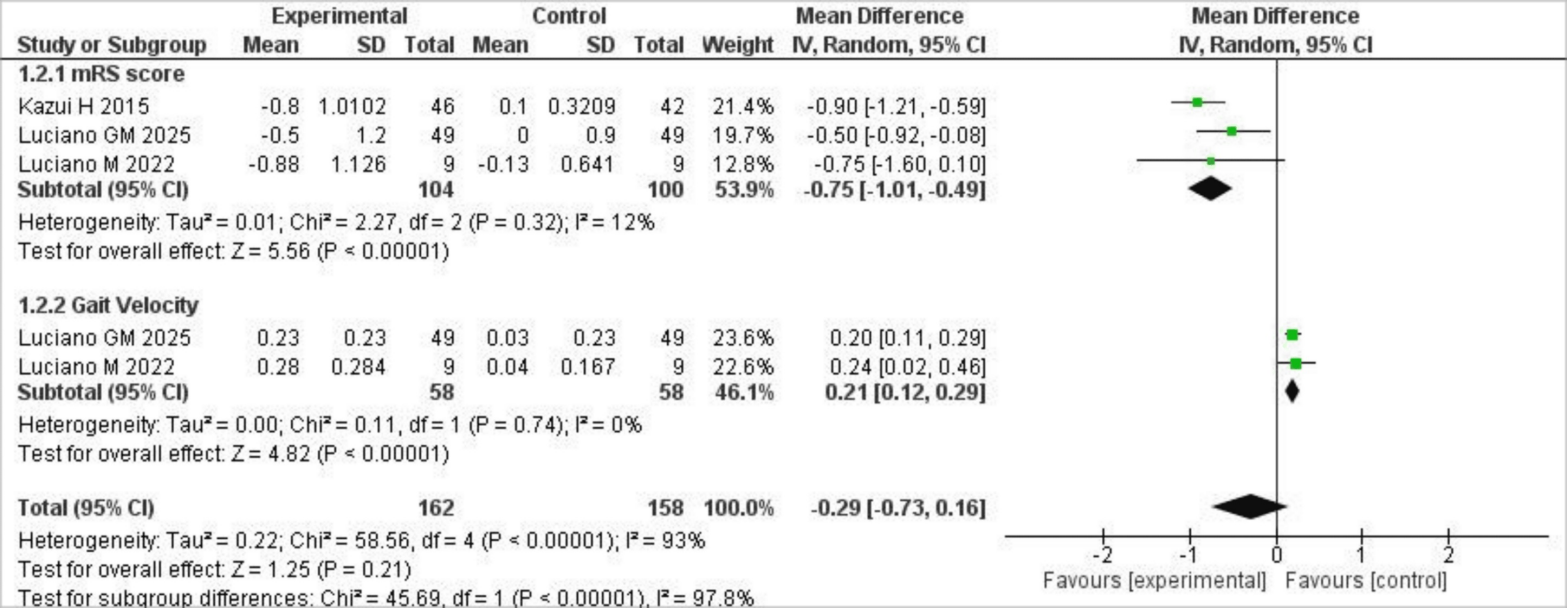
Forest plot of primary outcomes comparing CSF shunting versus placebo in patients with idiopathic normal pressure hydrocephalus. Forest plots comparing open shunting versus placebo for primary outcomes, including modified Rankin Scale (mRS) scores and gait velocity. Source: References [7,11,12].

Our primary outcomes consisted of changes in mRS scores from baseline, reported by all included trials, and changes in gait velocity from baseline, reported by only two trials. Our analysis showed that open shunting significantly improved mRS scores compared to placebo, with only low heterogeneity observed (I² = 12%; MD = -0.75; 95% CI (-1.01, -0.49), P < .00001). Similarly, our pooled analysis showed that gait velocity improved significantly with open shunting compared to placebo, with no heterogeneity observed (I² = 0%; MD = 0.21; 95% CI (0.12, 0.29), P < .00001). The forest plot for these results is shown below in Figure 3.

Secondary Outcomes

**Figure 4:**
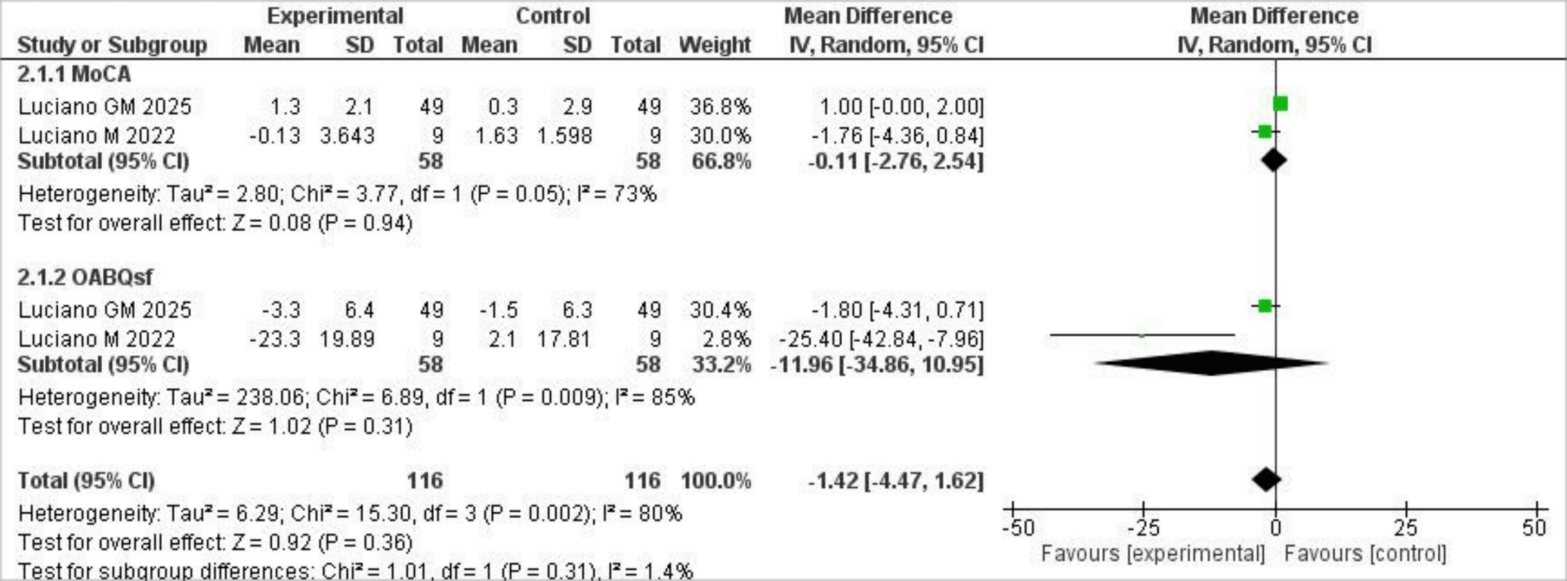
Forest plot of secondary outcomes comparing cerebrospinal fluid shunting versus placebo. Forest plots showing the effect of shunting on secondary outcomes, including Montreal Cognitive Assessment (MoCA) scores and Overactive Bladder Questionnaire Short Form (OAB-q SF) scores. Source: References [7,11,12].

Our secondary outcomes focused on MoCA scores and the Overactive Bladder Questionnaire Short Form (OAB-q SF), both of which were reported by only two trials. For MoCA scores, the analysis showed that the scores did not significantly improve with shunting compared to placebo, and moderate heterogeneity was observed, with I² being 73% (MD = -0.11; 95% CI (-2.76, 2.54), P = .94). Similarly, shunting was associated with improvement on the OABQsf compared to placebo. However, the results were not statistically significant, and high heterogeneity was observed, with I² at 85% (MD = -11.96; 95% CI (-34.86, 10.95), P = 0.31). The findings for this outcome are shown below in Figure 4.

Adverse Events

**Figure 5:**
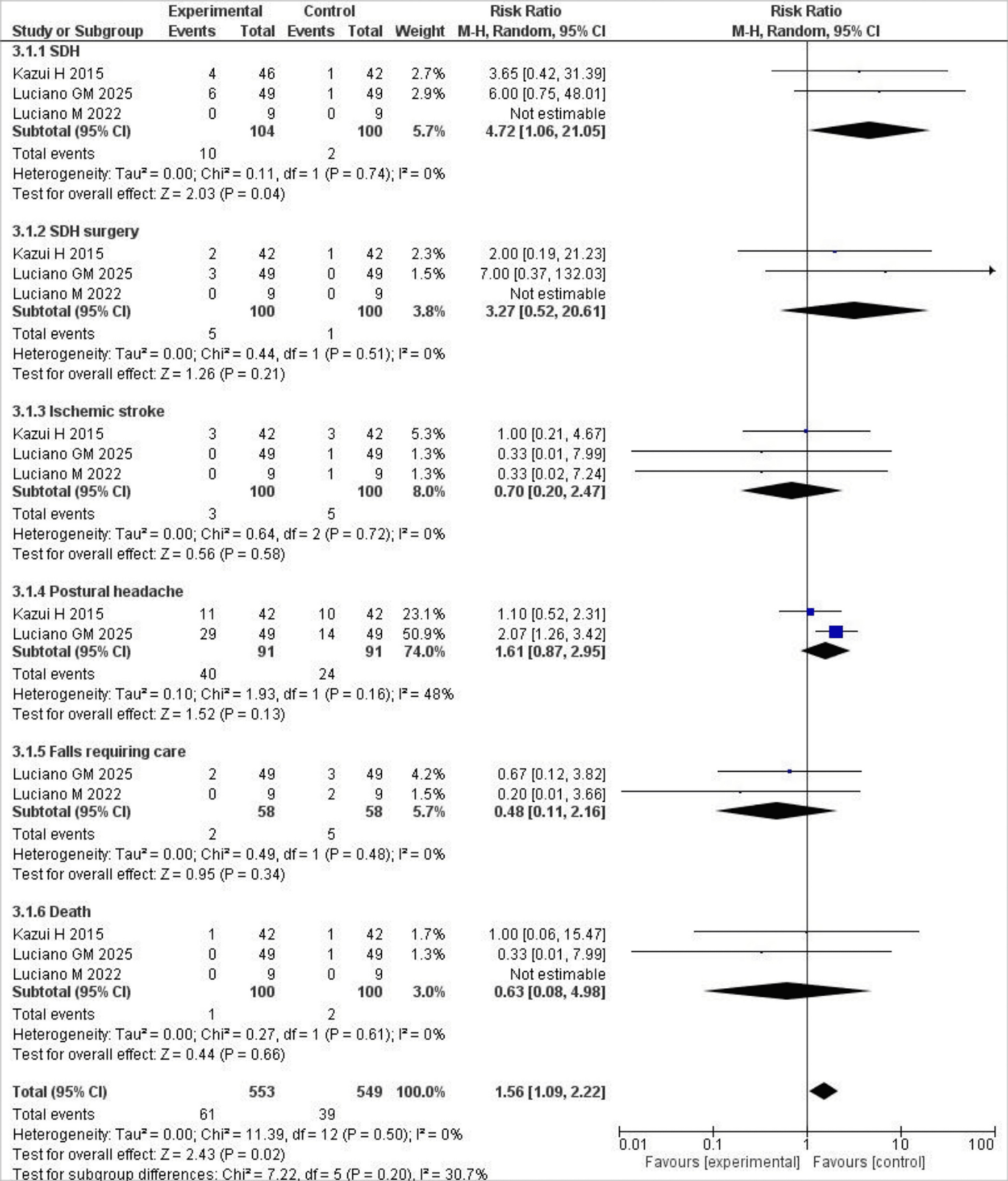
Forest plot of adverse events comparing cerebrospinal fluid shunting versus placebo. Forest plots comparing adverse events between the shunting and placebo groups, including subdural hematoma, postural headache, falls, ischemic stroke, and mortality. Source: References [7,11,12].

We also analyzed the differences between the two groups regarding common adverse events. For subdural hematoma, the risk was significantly higher in the experimental group than in the placebo group (RR = 4.72; 95% CI, 1.06-21.05; P = 0.04), with no heterogeneity. For SDH requiring surgery, the risk was again higher in the experimental group than in the placebo group, but the results were not significant (RR = 3.27; 95% CI, 0.52-20.67; P = 0.21). The risk of postural headaches was also higher in the experimental group, but again, the results were not significant (RR = 1.61; 95% CI, 0.87-2.95; P = 0.13). For falls that required care (RR = 0.48; 95% CI, 0.11-2.16; P = 0.13), ischemic stroke (RR = 0.70; 95% CI, 0.20-2.47; P = 0.58), and death (RR = 0.63; 95% CI, 0.08-4.98; P = 0.66), the results, although not significant, showed that the risk of these events was lower in the experimental group compared to placebo. No heterogeneity was observed for these three events. The results for adverse events are shown in the forest plot below (Figure 5).

Discussion

This study evaluated the effectiveness and safety of CSF shunting in individuals with iNPH based on evidence exclusively from RCTs, including preliminary pilot data and final trial results from the PENS study. The combined results show that CSF shunting correlates with overall functional improvement and gait performance, thereby suggesting potential efficacy as a treatment based on limited randomized evidence and informing the selection of the target patient population.

The most significant result of the present analysis showed a statistically significant positive effect on modified Rankin Scale (mRS) scores in patients receiving active shunting compared with placebo or delayed intervention. This improvement indicates a significant improvement in global functional autonomy rather than in the reduction of isolated symptoms. The results are consistent with past randomized and systematic review studies, which show reductions in disability with CSF diversion in iNPH [13,14]. Notably, the magnitude of the effect (MD -0.75) is close to a one-point shift on the ordinal mRS scale, which is usually considered clinically significant. This means that the perceived improvement can translate into patients’ increased ability to perform their daily tasks. The low heterogeneity also reinforces the validity of this observation, as it implies that functional benefit is consistent across study designs and shunt types.

There was a significant change in gait velocity, which was consistent after CSF shunting, and no heterogeneity was found across studies. This improvement (MD 0.21 m/s) exceeds generally accepted thresholds for clinically meaningful change: 0.10 m/s is considered meaningful, and 0.20 m/s is considered significant functional improvement. This suggests that the observed effect is likely to increase mobility and reduce the risk of falls. Gait impairment is often the most responsive component of the iNPH clinical triad, and improvement in gait parameters is often the best indicator of shunt responsiveness. The findings are consistent with current evidence of early and long-term improvements in gait following CSF diversion [15,16]. This effect is consistent across trials and thus also supports the clinical utility of gait measures as important endpoints for patient selection and postoperative assessments.

Unlike functional and gait outcomes, cognitive performance, measured by MoCA, did not exhibit statistically significant pooled improvement, and moderate heterogeneity was observed. Cognitive impairment in iNPH may be multifactorial and may reflect underlying neurodegenerative pathology or irreversible cortical alterations, which may restrict responsiveness to CSF diversion. This is consistent with previous research, which shows that although shunt surgery can improve specific cognitive functions, such as verbal memory, psychomotor speed, and selective executive functions, there is generally no enhancement in global cognitive functions. The improvement is highly inconsistent across patients [17]. Similarly, urinary symptoms assessed by the OAB-q SF did not show a statistically significant change and exhibited substantial heterogeneity across studies. This means that the response is variable and does not allow for a consistent treatment effect. This finding is consistent with reports that CSF shunting often helps relieve storage symptoms, such as urgency and urge incontinence, in some patients. Nevertheless, it may not eliminate voiding dysfunction or other urinary symptoms, leading to mixed outcomes across studies [18]. These findings suggest that cognitive and urinary outcomes might be less predictable with shunts and more difficult to measure in the long term or with assessment instruments that better reflect postoperative outcomes.

Subdural hematoma, a complication related to CSF overdrainage, was associated with an increased risk after CSF shunting and is well reported in the literature. Although the risk of subdural hematoma requiring surgical intervention was greater in the shunt group, the difference was not statistically significant, likely due to the small sample size. Other adverse outcomes, including postural headache, falls requiring care, ischemic stroke, and death, were not significantly different between groups. Overall, the safety profile generally aligns with that reported in prior literature, which emphasizes close monitoring of postoperative status and valve pressure [19,20,21].

Interestingly, trial design was also heterogeneous, including different control groups, such as placebo-controlled and delayed-intervention groups, and this may have contributed to clinical heterogeneity and impacted the pooled estimates.

Combined, these observations imply that CSF shunting could be beneficial in terms of functional status and gait in patients with iNPH, yet the evidence remains limited and must be viewed cautiously. The findings emphasize the importance of appropriate patient selection, case-by-case assessment of risks and benefits, and discussion of expected outcomes.

Limitations

There are several limitations to this meta-analysis. First, the number of eligible RCTs was small, limiting statistical power and precluding subgroup and sensitivity analyses. Second, variations in study design, shunt type (VP vs. LP), follow-up duration, and outcome measures, particularly for secondary outcomes, may have influenced the pooled estimates. Third, the limited number of included studies precluded a formal assessment of publication bias.

In addition, only two trials reported cognitive and urinary outcomes, reducing confidence in these pooled results. Furthermore, long-term follow-up beyond one year was limited, restricting conclusions regarding the durability of treatment effects. Finally, the included RCTs often enrolled highly selected patient populations under strict eligibility criteria, which may limit the external validity and generalizability of the findings to broader, real-world clinical populations.

Future Directions

Further study should emphasize large, multicenter randomized trials with standardized outcome measurements and extended follow-up periods to more comprehensively describe long-term effectiveness and safety. Predictors of shunt responsiveness should be considered in these studies, such as imaging biomarkers, physiological tests, and clinical phenotypes, to improve patient selection. Comparative trials of VP and LP shunts, valve technologies, and drainage modalities could potentially optimize outcomes. Also, the inclusion of patient-reported outcomes and quality-of-life measures would provide a more detailed evaluation of treatment benefit.

## Conclusions

This systematic review and meta-analysis show that CSF shunting improves functional status and gait performance in patients with iNPH, but at the expense of a higher risk of subdural hematoma. Less consistent and variable improvement was noted in cognitive and urinary outcomes. The results support the use of CSF shunting in selected patients with iNPH and indicate that careful risk assessment, patient education, and additional high-quality studies are required to streamline treatment plans.
